# Supplementary material for: Proximate composition, functional properties and quantitative analysis of benzoyl peroxide and benzoic acid in wheat flour samples: effect on wheat flour quality
Source: PeerJ. 2020 Mar 24;8:e8788. doi: 10.7717/peerj.8788 (PMC7100602; doi:10.7717/peerj.8788)
Supplement: Supplemental Information 1 [file peerj-08-8788-s001.docx]

| Time in hrs | BP (µg/g) | BA (µg/g) |
| --- | --- | --- |
| 0 | 29.5 | 2.84 |
| 1 | 16.5 | 3.55 |
| 2 | 5.7 | 5.64 |
| 3 | 4 | 7.14 |
| 4 | 3.5 | 8.9 |
| 5 | 2.5 | 9.41 |
| 6 | 1.2 | 10.31 |
| 7 | 0.9 | 11.84 |
| 8 | 0 | 13.5 |
| 9 | 0 | 13.75 |
| 10 | 0 | 13.7 |
| 11 | 0 | 13.65 |
| 12 | 0 | 13.66 |
