# Supplementary figures and images for: Proximate composition, functional properties and quantitative analysis of benzoyl peroxide and benzoic acid in wheat flour samples: effect on wheat flour quality
Source: PeerJ. 2020 Mar 24;8:e8788. doi: 10.7717/peerj.8788 (PMC7100602; doi:10.7717/peerj.8788)

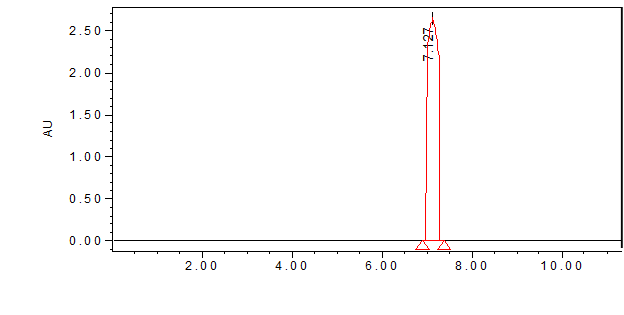

Supplement: Supplemental Information 2 [file peerj-08-8788-s002.png]
